# The Joint Association of Small for Gestational Age and Nighttime Sleep with Blood Pressure in Childhood

**DOI:** 10.1038/s41598-018-27815-1

**Published:** 2018-06-25

**Authors:** Hongjian Wang, Noel Mueller, Guoying Wang, Xiumei Hong, Ting Chen, Yuelong Ji, Colleen Pearson, Lawrence J. Appel, Xiaobin Wang

**Affiliations:** 1 Department of Cardiology, State Key Laboratory of Cardiovascular Disease, National Clinical Research Center of Cardiovascular Diseases, Fuwai Hospital, National Center for Cardiovascular Diseases, Chinese Academy of Medical Sciences and Peking Union Medical College, Beijing, China; 2 Center on the Early Life Origins of Disease, Department of Population, Family and Reproductive Health, Johns Hopkins University Bloomberg School of Public Health, Baltimore, MD USA; 3 Department of Epidemiology, Johns Hopkins Bloomberg School of Public Health, Baltimore, Maryland USA; 4 Welch Center for Prevention, Epidemiology and Clinical Research, Johns Hopkins University, Baltimore, Maryland USA; 5 Department of Pediatrics, Boston University School of Medicine and Boston Medical Center, Boston, Massachusetts, USA; 6 Division of General Pediatrics & Adolescent Medicine, Department of Pediatrics, Johns Hopkins University School of Medicine, Baltimore, MD USA

## Abstract

Children born small for gestational age (SGA) are more likely to develop high blood pressure. In prior studies, longer sleep duration is associated with lower BP, and SGA is associated with shorter sleep duration in childhood. We investigated whether sleep duration in early childhood modifies the association between SGA and higher childhood SBP in 1178 children recruited at birth and followed up to age 9 years. We ascertained birthweight and gestational age from medical records. We derived child sleep duration from maternal questionnaire interview. We calculated child SBP percentile according to U.S. reference data. We defined elevated SBP as SBP ≥75^th^ percentile. In this sample, 154 (13.1%) children were born SGA. Children born SGA had higher SBP percentiles and higher risk of elevated SBP. Among children born SGA, those in the highest compared to the lowest tertile for sleep had a 12.28 lower (−22.00, −2.57) SBP percentile and 0.44 (0.25 to 0.79) times lower risk of developing elevated SBP. Our data are consistent with an interaction between SGA and sleep duration on childhood elevated SBP (P_interaction_ = 0.0056). In conclusion, in this prospective birth cohort, longer sleep duration in early childhood may mitigate the blood pressure-raising effect of being born small.

## Introduction

Worldwide, high blood pressure is the leading cause of preventable mortality^1^. Blood pressure in childhood has been shown to track into adulthood, increasing risk for cardiovascular disease morbidity and mortality throughout life^2^. It is now recognized that the antecedents to childhood high blood pressure originate *in utero* and that efforts should focus on primordial prevention in mothers and primary prevention in the pediatric age group^3,4^.

Being born small for gestational age (SGA) is a marker of intrauterine growth-restriction. Likely due to inadequate organ development *in utero*, infants born SGA are known to be at higher risk of developing high blood pressure^5^ and atherosclerotic cardiovascular disease later in life^6^. Small size at birth is also associated with shorter sleep duration in early childhood^7^ and lower sleep efficiency after adjustment for gestational age and sex^8^. In separate studies, longer sleep is associated with lower blood pressure^9,10^. This constellation of findings raises an important research question—can longer sleep duration in childhood help lower high blood pressure in children born SGA?

In a well-established U.S., predominantly urban, low income minority prospective birth cohort, a population know to be at high risk of hypertension, we aimed to examine the association of child sleep duration and child BP according to the level of birthweight for gestational age (BW-GA) category, and to test the hypothesis that longer sleep duration mitigates the positive association between SGA and systolic BP (SBP) later in childhood. The findings from our study have potential implications for primary prevention of hypertension and its consequences among high risk children during early developmental windows, as sleep duration represents a potentially modifiable risk factor.

## Results

The final analytic sample comprised 1178 children, including 488 Black (41.4%) and 217 Hispanic (18.4%) children. In Table 1, we present characteristics of mothers and children in our study according to BW-GA categories and sleep duration tertiles. A total of 335 (28.4%) children had elevated SBP (defined in this study as SBP ≥ 75^th^ percentile) at their final study visit between the 3 and 9 years of age.

**Table 1:** Characteristics of mothers and children in the Boston Birth Cohort (BBC) according to birth weight for gestational age categories and nighttime sleep duration tertiles (from lowest tertile [T1] to highest tertile [T3]).

**Variables**	**Small for Gestational Age**			**Appropriate for Gestational Age**			**Large for Gestational Age**		
	**Nighttime Sleep Duration Tertiles**			**Nighttime Sleep Duration Tertiles**			**Nighttime Sleep Duration Tertiles**		
	**Short (T1)**	**Medium (T2)**	**Long (T3)**	**Short (T1)**	**Medium (T2)**	**Long (T3)**	**Short (T1)**	**Medium (T2)**	**Long (T3)**
Number	39	60	55	305	294	304	42	48	31
***Maternal Characteristics***									
Race, n. (%)									
Black	21 (53.9)	27 (45.0)	25 (45.5)	141 (46.2)	117 (39.8)	106 (34.9)	20 (47.6)	22 (45.8)	9 (29.0)
Hispanic	5 (12.8)	9 (15.0)	15 (27.3)	52 (17.1)	60 (20.4)	57 (18.8)	7 (16.7)	4 (8.3)	8 (25.8)
Haitian	6 (15.4)	9 (15.0)	7 (12.7)	74 (24.3)	63 (21.4)	86 (28.3)	9 (21.4)	13 (27.1)	6 (19.4)
Other	7(18.0)	15 (25.0)	8 (14.6)	38 (12.5)	54 (18.4)	55 (18.1)	6 (14.3)	9 (18.8)	8 (25.8)
Pre-pregnancy BMI, kg/m^2^	25.1 (5.9)	25.7 (8.5)	25.4 (6.4)	27.0 (6.6)	27.4 (7.0)	26.2 (5.9)	30.4 (8.5)	29.1 (6.1)	29.7 (6.3)
Maternal Diabetes n. (%)	4 (10.3)	6 (10.0)	3 (5.5)	29 (9.5)	34 (11.6)	32 (10.5)	11 (26.2)	18 (37.5)	8 (25.8)
Maternal Hypertensive Disorder, n. (%)	9 (23.1)	13 (21.7)	17 (30.9)	49 (16.1)	46 (15.7)	50 (16.5)	9 (21.4)	10 (20.8)	5 (16.1)
***Children Characteristics***									
Age, years	6.9 (2.0)	7.4 (2.1)	7.2 (2.0)	6.8 (2.1)	6.8 (2.1)	7.0 (2.1)	7.1 (2.0)	6.6 (2.2)	7.1 (2.3)
Boys, n. (%)	15 (38.5)	33 (55.0)	30 (54.6)	158 (51.8)	149 (50.7)	154 (50.7)	21 (50.0)	22 (45.8)	16 (51.6)
Birthweight (g)	2284.1 (518.2)	2354.0 (420.1)	2222.5 (573.7)	2891.1 (760.6)	2934.5 (778.0)	2933.6 (709.4)	3804.0 (698.4)	3935.5 (614.0)	3994.4 (485.4)
Low birthweight n. (%)	23 (59.0)	38 (63.3)	35 (63.6)	68 (22.3)	59 (20.1)	62 (20.4)	2 (4.8)	2 (4.2)	1 (3.2)
Gestational age(week)	38.1 (2.3)	38.6 (2.0)	37.8 (3.0)	37.6 (3.7)	37.8 (3.8)	37.8 (3.4)	37.9 (3.1)	38.4 (2.9)	38.5 (2.2)
Preterm birth, n. (%)	8 (20.5)	8 (13.3)	14 (25.5)	78 (25.6)	75 (25.5)	81 (26.6)	13 (31.0)	11 (22.9)	7 (22.6)
BMI z-score	0.26 (1.34)	0.40 (1.42)	0.10 (1.55)	0.80 (1.17)	0.80 (1.25)	0.82 (1.28)	1.53 (0.88)	1.25 (1.30)	1.15 (0.99)
Weight z-score	0.02 (1.29)	0.22 (1.30)	-0.16 (1.75)	0.82 (1.20)	0.88 (1.20)	0.86 (1.22)	1.58 (0.91)	1.57 (1.07)	1.24 (1.06)
SBP percentiles	67.8 (25.5)	57.3 (25.3)	56.1 (22.0)	55.7 (25.4)	59.3 (24.2)	55.5 (25.1)	55.4 (27.1)	53.7 (23.9)	52.6 (25.6)
Elevated SBP n. (%)	20 (51.3)	19 (31.7)	12 (21.8)	82 (26.9)	91 (31.0)	84 (27.6)	12 (28.6)	9 (18.8)	6 (19.4)

BMI: Body Mass Index; Data are shown as mean (standard deviation, SD) or *n* (%). Sleep duration tertiles were specific for age (based on sleep questionnaire visit age) and sex: tertile 1 (T1) represents “short” sleep duration; tertile 2 (T2) represents “medium” sleep duration; and tertile 3 (T3) represents “long” sleep duration.

**Figure 1:**
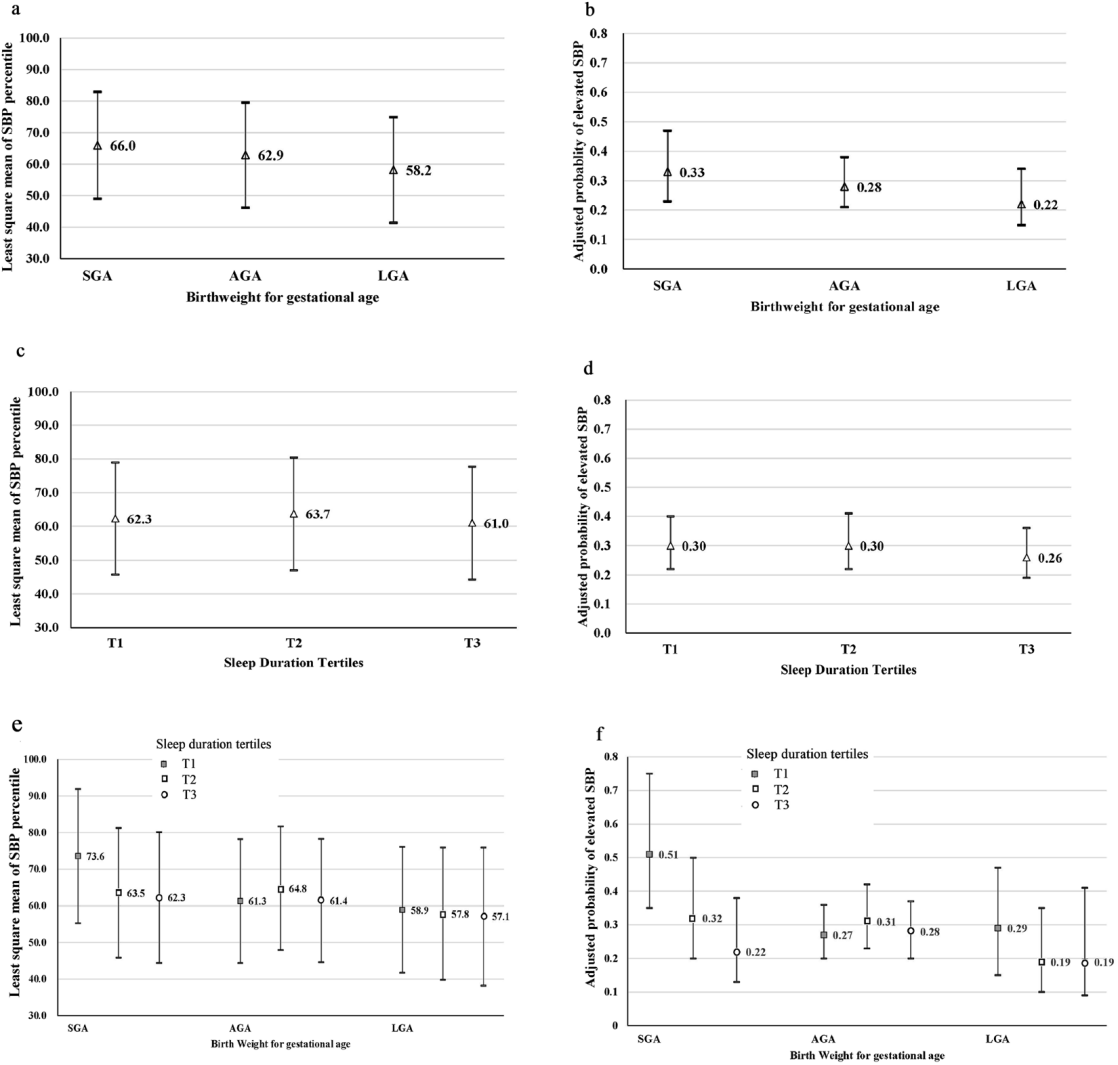
Individual and combined associations of birth weight for gestational age and child sleep duration with child SBP percentile and elevated SBP (SBP ≥75th percentile) in the Boston Birth Cohort (BBC). (**a**) The y-axis represents least square means and 95% CI of child SBP percentiles, as estimated using a generalized linear model with adjustment for sex, age, race/ethnicity (Black, Hispanic, Haitian, others), maternal pre-pregnancy BMI (continuous), maternal hypertensive disorder (yes vs. no), maternal diabetes (yes vs. no) and sleep duration. (**b**) The y-axis represents adjusted probabilities and 95%CI of child elevated SBP, as estimated using a Poisson regression model with adjustment for the previously mentioned covariates (**a**). (**a,b**) The X-axis represents birth weight for gestational age categories; SGA: small for gestational age; LGA: large for gestational age; AGA: appropriate for gestational age. (**c**) The y-axis represents least square means and 95% CI of child SBP percentiles, as estimated using a generalized linear model with adjustment for sex, age, race/ethnicity (Black, Hispanic, Haitian, others), maternal pre-pregnancy BMI (continuous), maternal hypertensive disorder (yes vs. no), maternal diabetes (yes vs. no) and birth weight for gestational age. (**d**) The y-axis represents adjusted probabilities and 95% CI of child elevated SBP, as estimated using a Poisson regression model with adjustment for the previously mentioned covariates (**c**). (**c,d**) The X-axis represents sleep duration categories; Sleep duration tertiles were specific for age (based on sleep questionnaire visit age) and sex: tertile 1(T1) represents “short” sleep duration; tertile 2 (T2) represents “medium” sleep duration; and tertile 3 (T3) represents “long” sleep duration. (**e**) The y-axis represents least square means and 95%CI of child SBP percentiles, as estimated using a generalized linear model with adjustment for sex, age, race/ethnicity (Black, Hispanic, Haitian, others), maternal pre-pregnancy BMI (continuous), maternal hypertensive disorder (yes vs. no) and maternal diabetes (yes vs. no). P for interaction between birthweight for gestational age and sleep duration on SBP percentiles: 0.087 (**f**) The y-axis represents adjusted probabilities and 95%CI of child elevated SBP, as estimated using a Poisson regression model with adjustment for the previously mentioned covariates (e). P for interaction between birthweight for gestational age and sleep duration on elevated SBP: 0.0056.

**Figure 2:**
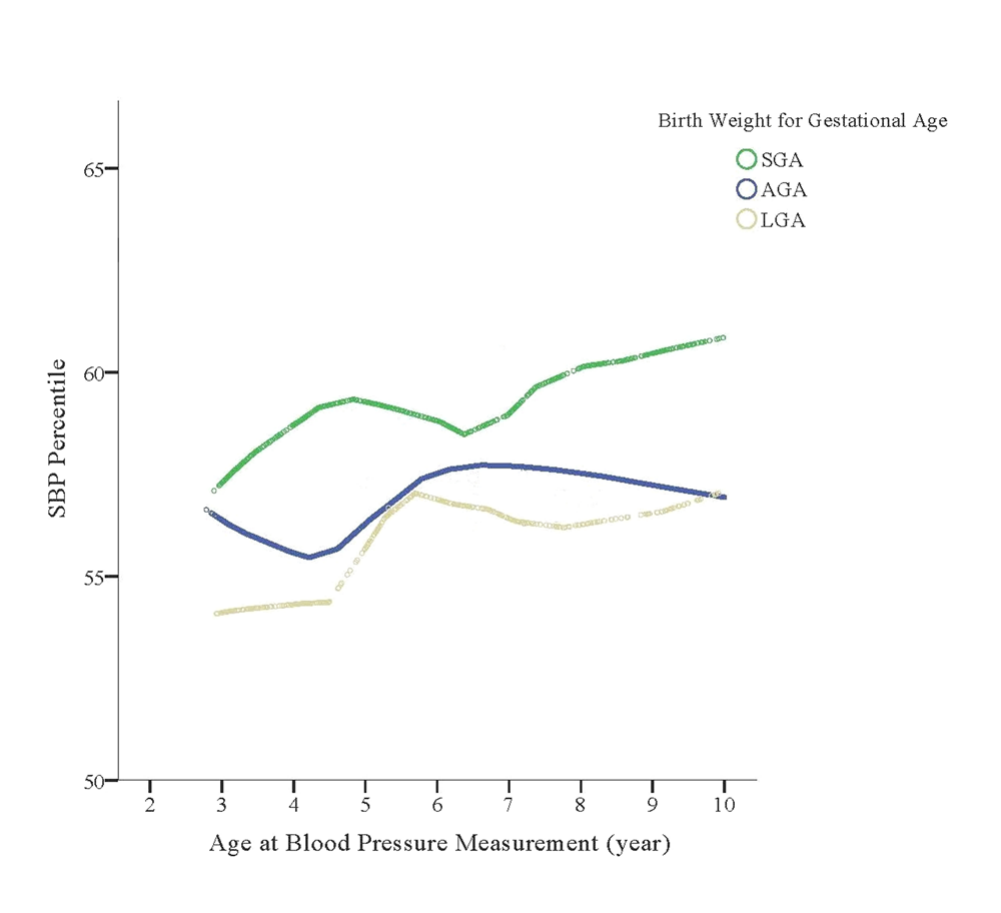
Tracking of blood pressure in early childhood according to birth weight for gestational age categories. Y-axis represents the mean of SBP percentiles and the X-axis represents the age at blood pressure measurement; SGA: small for gestational age; LGA: large for gestational age; AGA: appropriate for gestational age.

Children born SGA had higher SBP percentiles and higher risk of elevated SBP, before and after adjusting for covariates (Table 2; Fig. 1a,b). This association was consistent from 3 to 9 years of age (Fig. 2). Overall, childhood sleep duration was not associated with child SBP percentile or elevated SBP (Table 2; Fig. 1c,d). However, we found evidence (Fig. 1e,f and Table 3) of an interaction between childhood sleep duration and BW-GA on offspring SBP percentiles (P_interaction_ = 0.087) and risk of elevated SBP (P_interaction_ = 0.0056).

**Table 2:** The individual associations of birth weight for gestational age and child sleep duration with child SBP percentile and elevated SBP (SBP ≥75%) in children from the Boston Birth Cohort (BBC).

	N	Child SBP percentile			Child elevated SBP		
		Mean (SD)	Model 1	Model 2	Case, No (%)	Model 1	Model 2
			ß (95%CI)	ß (95%CI)		RR (95%CI)	RR (95%CI)
***Birthweight for gestational age*** ^a^							
LGA	121	54.0 (25.3)	0		27 (22.3)	1	
AGA	903	56.8 (25.0)	4.70 (−0.09,9.50)	5.84 (1.08,10.60)*	257 (28.5)	1.41 (1.00,2.00)	1.51 (1.06,2.14)*
SGA	154	59.5 (24.6)	7.78 (1.74,13.82)*	10.13 (4.08,16.19)*	51 (33.1)	1.65 (1.10,2.47)*	1.86 (1.23,2.80)^†^
BW-GA			−1.44 (−2.82, −0.05)*	−2.09 (−3.49, −0.69)^†^		0.92 (0.84,1.00)	0.89 (0.82,0.98)*
***Sleep duration tertiles*** ^b^							
Short (T1)	386	56.9 (25.8)	0		114 (29.5)	1	
Medium (T2)	402	58.4 (24.4)	1.40 (−2.06,4.86)	1.81 (−1.62,5.24)	119 (29.6)	1.01 (0.82,1.26)	1.03 (0.83,1.27)
Long (T3)	390	55.3 (24.7)	−1.33 (−4.82,2.16)	−0.99 (−4.45,2.47)	102 (26.2)	0.90 (0.72,1.13)	0.92 (0.73,1.15)
1-hour increment in sleep duration			−0.78 (−1.87,0.31)	−0.62 (−1.71,0.46)		0.95 (0.88,1.02)	0.96 (0.89,1.03)

Model 1: Adjusted by sex, age, race/ethnicity (Black, Hispanic, Haitian, others), maternal pre-pregnancy BMI (continuous), maternal hypertensive disorder (yes vs. no) and maternal diabetes (yes vs. no); Model 2: Model 1 with children BMI z score; *P < 0.05; ^†^P < 0.01; ^a^additional adjustment for sleep duration; ^b^additional adjustment for birth weight for gestational age; BW-GA: Birth weight for gestational age.

**Table 3:** The combined association of birth weight for gestational age and child sleep duration on child SBP percentile and elevated SBP (SBP ≥75%) in children from the Boston Birth Cohort (BBC)

	**N**	**Child SBP percentile**			**Child elevated SBP**		
		**Mean (SD)**	**Model 1**	**Model 2**	**Case, No (%)**	**Model 1**	**Model 2**
			**ß (95%CI)**	**ß (95%CI)**		**RR (95%CI)**	**RR (95%CI)**
***Combined effect of birth weight for gestational age and sleep duration on blood pressure***							
***SGA***							
Short (T1)	39	67.8 (25.5)	0		20 (51.3)	1	
Medium (T2)	60	57.3 (25.3)	−10.07 (−20.00, −0.15)*	−9.44 (−19.31,0.43)	19 (31.7)	0.64 (0.40, 1.02)	0.64 (0.40,1.04)
Long (T3)	55	56.1 (22.0)	−11.34 (−21.44, −1.24)*	−9.88 (−19.94,0.17)	12 (21.8)	0.43 (0.24,0.76)^†^	0.45 (0.25,0.79)^†^
***AGA***							
Short (T1)	305	55.7 (25.4)	−12.29 (−20.51, −4.08)^†^	−12.98 (−21.21, −4.75)^†^	82 (26.9)	0.53 (0.37,0.75)^†^	0.51 (0.35,0.72)^†^
Medium (T2)	294	59.3 (24.2)	−8.79 (−17.03, −0.55)*	−9.11 (−17.36, −0.86)*	91 (31.0)	0.61 (0.43,0.86)^†^	0.59 (0.42,0.83)^†^
Long (T3)	304	55.5 (25.1)	−12.16 (−20.37, −3.94)^†^	−12.77 (−21.00, −4.54)^†^	84 (27.6)	0.56 (0.39,0.79)^†^	0.53 (0.37,0.76)^†^
***LGA***							
Short (T1)	42	55.4 (27.1)	−14.69 (−25.52, −3.86)^†^	−16.86 (−27.69, −6.03)^†^	12 (28.6)	0.49 (0.27,0.87)*	0.43 (0.24,0.77)^†^
Medium (T2)	48	53.7 (23.9)	−15.75 (−26.23, −5.28)^†^	−17.35 (−27.81, −6.89)^†^	9 (18.8)	0.35 (0.18,0.67)^†^	0.31 (0.16,0.60)^†^
Long (T3)	31	52.6 (25.6)	−16.53 (−28.20, −4.86)^†^	−17.64 (−29.26, −6.03)^†^	6 (19.4)	0.38 (0.17, 0.81)*	0.35 (0.16,0.76)^†^
***Stratified by birth weight for gestational age***							
***SGA***							
Short (T1)	39	67.8 (25.5)	0		20 (51.3)	1	
Medium (T2)	60	57.3 (25.3)	−10.43 (−19.91, −0.95)*	−9.59 (−19.11, −0.06)*	19 (31.7)	0.62 (0.37,1.03)	0.66 (0.39,1.09)
Long (T3)	55	56.1 (22.0)	−12.28 (−22.00, −2.57)*	−11.35 (−21.13, −1.57)*	12 (21.8)	0.44 (0.25,0.79)^†^	0.47 (0.27,0.82)*
1-hour increment in sleep duration			−3.74 (−7.12, −0.36)*	−3.42 (−6.83, −0.02)*		0.76 (0.63,0.91)^†^	0.78 (0.65,0.93)^†^
***AGA***							
Short (T1)	305	55.7 (25.4)	0		82 (26.9)	1	
Medium (T2)	294	59.3 (24.2)	3.49 (−0.48,7.45)	3.86 (−0.07,7.78)	91 (31.0)	1.15 (0.90,1.48)	1.17 (0.91,1.50)
Long (T3)	304	55.5 (25.1)	0.17 (−3.77, 4.12)	0.23 (−3.66,4.13)	84 (27.6)	1.06 (0.82,1.37)	1.06 (0.82,1.37)
1-hour increment in sleep duration			−0.48 (−1.68,0.72)	−0.40 (−1.58,0.79)		0.99 (0.91,1.07)	0.99 (0.92,1.07)
***LGA***							
Short (T1)	42	55.4 (27.1)	0	0	12 (28.6)	1	
Medium (T2)	48	53.7 (23.9)	−2.39 (−12.21, 7.43)	−1.98 (−11.80,7.85)	9 (18.8)	0.64 (0.30,1.38)	0.64 (0.29,1.40)
Long (T3)	31	52.6 (25.6)	−0.25 (−11.20, 10.70)	0.34 (−10.65,11.33)	6 (19.4)	0.86 (0.38,1.94)	0.86 (0.38,1.93)
1-hour increment in sleep duration			0.18 (−3.82, 4.17)	0.35 (−3.64, 4.35)		0.89 (0.66,1.20)	0.89 (0.65,1.21)

Model 1: adjusted for sex, age, race/ethnicity (Black, Hispanic, Haitian, others), maternal pre-pregnancy BMI (continuous), maternal hypertensive disorder (yes vs. no) and maternal diabetes (yes vs. no); Model 2: Model 1 with children BMI z score; *P < 0.05; ^†^P < 0.01; P for interaction between birthweight for gestational age and sleep duration on elevated SBP: 0.0056. P for interaction between birthweight for gestational age and sleep duration on SBP percentiles: 0.087.

Among SGA children, those in the lowest sleep duration tertile (compared to those in the highest sleep duration tertile) had more than two times higher risk of developing elevated SBP [relative risk (RR) = 2.25; 95% confidence interval (CI): 1.27–3.98]. SGA children in the highest sleep tertile had 12.28 percentile (95% CI: −22.00, −2.57) lower SBP and a 56% lower risk (RR = 0.44, 95% CI: 0.25, 0.79) of developing elevated SBP compared to those in the lowest sleep tertile. As a result, the probability of elevated BP in SGA children that were in the highest sleep tertile was indistinguishable from children born appropriate for gestational age (AGA) or large for gestational age (LGA) (Fig. 1e,f). Sleep duration was not associated with BP in children born AGA or LGA (Table 3).

Modelling sleep continuously, a 1-hour increment in sleep duration for children born SGA is associated with a 3.74 percentile decrease (95%CI: −7.12, −0.36) in SBP percentile and 0.76 (95% CI: 0.63, 0.91) times lower risk of developing elevated SBP (Table 3). Associations were not materially changed after additional adjustment for childhood body mass index (BMI) status (Tables 2 and 3, Model 2) or socioeconomic indicators (maternal education and the total year household income)(Supplementary Tables S1 and S2). Corresponding analyses on diastolic BP (DBP) (Supplementary Tables S3 and S4) found that the individual and combined associations of sleep duration and BW-GA on DBP were not significant.

The associations described above also did not differ appreciably when we restricted analyses to participants who have 2 or more BP measurements during childhood (data not shown) nor were they materially different when we restricted analyses to sleep duration ascertained between 4 to 9 years of age (Supplementary Tables S5 and S6). Furthermore, the associations were not markedly different when we used a cutoff of SBP ≥90th percentile or ≥120 mmHg, or a cutoff of SBP/DBP ≥90th percentile or ≥120/80 mmHg^11^ (Supplementary Tables S7 and S8), or when we used alternative national BW-GA standards (data not shown).

## Discussion

In this prospective U.S. birth cohort study, our findings provide further support for the hypothesis that being born SGA is associated with higher SBP throughout childhood. Adding to this body of literature, we demonstrate for the first time that longer nighttime sleep duration in early childhood significantly attenuated the association between SGA and higher SBP, such that the SBP of SGA children in the highest tertile of sleep duration was not appreciably different from children born AGA or LGA.

Our findings on SGA and BP are consistent with decades of research, starting with Barker and colleagues who showed that markers of intrauterine growth restriction are associated with higher SBP in children (at age 10) and adults (at age 36)^12^. A more contemporary prospective birth cohort found an inverse relationship between birth weight and BP measured in infants during the first week of life^13^. Potential mechanisms underlying these associations include inadequate *in utero* development of key organs, including the kidneys, and structural changes to blood vessels among children born SGA^14^. Studies have also shown that low birth weight is associated with a reduced nephron complement^15^ and a reduced nephron number contributes to the genesis of hypertension^16^. Rosenfeld *et al*. further implicated kidney dysfunction when they found that very-low-birth-weight preterm infants had reduced estimated glomerular filtration rate and higher SBP at 3 years^17^.

It previously reported that rapid post-natal weight gain in SGA children was associated with high BP^18^. In our cohort, postnatal weight gain per year was much higher in SGA than in LGA children. When we additionally adjusted for weight gain per year, the association of BW-GA with SBP was attenuated and no longer significant, suggesting that weight gain may in part mediate the association. However, the combined association of BW-GA and sleep duration with SBP remained similar after adjusting for weight gain. Similar to other studies, BMI z-score is strongly associated with SBP in our cohort. However, when our analysis was stratified by BW-GA groups, there was no significant association of BMI z-score on BP in the LGA and SGA groups. We speculate that the major reason of higher prevalence of elevated BP in children born SGA was due to their inadequate organ development or programming in utero.

Evidence linking sleep duration with childhood BP has thus far been mixed^6,9,19–22^. Some studies have found short sleep duration is associated with higher blood pressure^9,19,20^. However, other studies observed no relationship between sleep duration and BP^20,22,23^. This mixed finding could be due to failure to account for SGA as a potential effect modifier. In addition to being linked with higher blood pressure, SGA has also been associated with shorter sleep duration and quality^7,8^, which may exacerbate metabolic dysfunction and blood pressure regulation^24–28^.

To the best of our knowledge, none of these previous studies evaluated whether the association of sleep duration with childhood blood pressure could be modified by fetal growth restriction (i.e. being born SGA). For the first time, our findings suggest that greater sleep duration in early childhood may mitigate the elevated SBP associated with being born SGA.

Our study has some limitations. First, there may be bias and measurement error in the maternal reporting of child sleep. There is a moderate correlation between self-reported sleep duration and actigraphy-assessed time spent asleep (ρ = 0.43) and time spent in bed (ρ = 0.48)^29^. However, sleep questionnaires, by design, may better reflect long-term sleep patterns, which may not be well-captured by actigraphy. We speculate that sleep measurement error is likely non-differential, thus, may bias our results towards null. Our study focused on nighttime sleep, and we did not have data on napping. This may have affected our sleep duration calculation, especially for younger ages. Nevertheless, when we restricted analyses to sleep data ascertained between ages 4–9 years the observed associations persisted. Second, while control for physical activity did not alter the observed associations, our assessment of physical activity was based on a single question: “About how physically active is your child compared to other children his/her age? Would you say about the same, a lot less, a little less, a little more physically active, or a lot more?” Third, the BP measurements were derived from pediatric well-child visits and may be prone to measurement errors. Yet when we restricted analyses to participants who have 2 or more BP measurements, the associations did not differ appreciably. Forth, our findings in this urban, low-income minority U.S. population may be relevant to other disadvantaged populations, but caution is needed to generalize our findings to populations with different characteristics. A fifth limitation is that only a sub-set of the Boston Birth Cohort (BBC) participants had data on sleep and blood pressure in childhood. While we cannot exclude the possibility of selection bias, when we compared the 1178 participants included in our final analysis to those 708 participants that did not have data on key covariates, we did not find material differences (Supplementary Table S9). Another potential limitation is that our definition of elevated BP (SBP ≥75%) may include children with high normal BP (75th up to 95th percentiles). Although this group would still be considered having normal BP clinically, they are at higher risk of future hypertension given the strong tracking of SBP across ages in this population^2^. Finally, as this is an observational study, we cannot rule out the possibility of unmeasured or residual confounding. Our findings should be regarded as hypothesis generating and additional studies are needed to confirm our findings and to elucidate the underlying mechanisms.

### Perspectives

To our knowledge, this is the first prospective birth cohort study to evaluate the independent and joint associations of SGA and early childhood sleep duration on risk of elevated BP in childhood. The ascertainment prenatal data, along with our longitudinal assessment of childhood blood pressure data and postnatal modifiers, such as sleep duration, uniquely enabled us to address this important research question in our birth cohort of children at high risk for elevated BP. Our findings lend further support on the persistence of elevated BP among contemporary multi-ethnic children born SGA, and raise the possibility that longer sleep duration may help mitigate future risk of high blood-pressure associated with SGA. Our findings lay the groundwork for future studies to dissect the etiology of this relationship in the SGA setting and determine whether or not postnatal sleep manipulations can influence BP in SGA-born children.

## Methods

### Study Participants

Participants for this study were from the Boston Birth Cohort (BBC). The BBC has registered with clinicaltrials.gov (https://clinicaltrials.gov/ct2/show/NCT03228875). In 1998, the BBC began enrollment of mother-child dyads at the Boston Medical Center (BMC). The cohort comprises a largely urban and low-income minority population, many of which are preterm, low birth-weight babies^30^. Eligible for inclusion in the initial enrollment were all mothers who delivered preterm (<37 weeks) or low birthweight (<2500 g) infants; mothers who delivered at term (≥37 weeks) and normal birthweight (>2500 g) infants were matched by maternal age and parity and included at a ratio of 1:2. Exclusion criteria for the initial enrollment included multiple-gestation pregnancies, pregnancies from *in vitro* fertilization, deliveries by mother with trauma, and infants with major birth defects^31^. At enrollment (between 1998–2012) a standardized questionnaire was administered with mothers who provided informed consent, within 2 to 3 days of delivery, to ascertain information on social, demographic and environmental factors.

The participation rate for initial enrollment and postnatal follow-up among eligible participants approached by the research staff was >90%^30^. All children enrolled in the BBC and who received primary care at the BMC were eligible for follow-up between 2003–2014. There was no difference in maternal demographic characteristics and birth outcomes between those enrolled in follow-up and those not^30^. We used a standardized questionnaire to evaluate postnatal sleep duration, feeding/diet, and home environment at follow-up visit. The Institutional Review Boards of Boston University Medical Center and Johns Hopkins Bloomberg School of Public Health approved the study protocol and consent. The study and methods were performed in accordance with the relevant guidelines and regulations.

In Supplementary Figure S1, we illustrated how participants were selected for this analysis. There were 3098 mother-infant pairs enrolled in the postnatal follow-up study at BMC at the time of the study. Of those, 1886 mother-children pairs completed at least one well-child visit with BP measurements at age 3 to 9 years. We further restricted the current sleep analysis to the 1178 children whose parents had completed a sleep questionnaire (follow-up visit, all sleep data was ascertained between 1 and 9 years of age) before or at the time of BP measurement (well-child visit), and whose mothers provided data on pre-pregnancy body mass index (BMI), diabetes (either pre-existing or gestational diabetes), and hypertensive disorders in pregnancy. Compared to the 708 mother-child pairs excluded from our analytic sample, due to the missing values on key covariates, the children of the 1178 pairs included in the analyses were, on average, older, and more likely to be boys and to be born to mothers who had diabetes. Other maternal demographic characteristics, birth outcomes were comparable between children included and excluded from the study (Supplementary Table S9).

### Sleep duration

We ascertained average sleep duration from four questions that asked parents about their child’s typical bedtime and wake-up time on weekdays and weekend or vacation days: “What time does your child usually go to bed (fall asleep) during week?” “What time does your child usually go to bed (fall asleep) on the weekend or vacation?” “What time does your child usually get out of bed (wake up) on weekday morning?” “What time does your child usually get out of bed (wake up) on weekend or vacation morning?”. We calculated total sleep time (TST) as the duration from bedtime to wake-up time on weekdays and weekend or vacation days, and mean sleep duration as:

$$\frac{TST\,weekday\times 5+TST\,weekend\times 2}{7}$$

### Anthropometric outcomes and blood pressure

Clinical staff measured child weight, height and BP during well-child visits, as documented in the electronic medical records. We calculated BMI as weight in kilograms divided by height in meters squared (kg/m^2^). We calculated height, weight and BMI *z*-scores using U.S. reference data^32^. We focused on childhood SBP as our primary outcome variable, rather than DBP because SBP is a better predictor of later cardiovascular outcomes and because it is more accurately measured^33,34^.

Child BP was measured at the BMC in a quiet room, using an appropriate size cuff, with clinical staff measuring at the right brachial artery using the validated automatic sphygmomanometer Masimo Set (2003–2008: the Welch Allyn 420 Spot Vital Signs monitor; 2008–2014, the Welch Allyn 45MT0 Spot Vital Signs LXi monitor). We calculated BP percentile using a U.S. national reference for age, sex and height^35^. Consistent with a definition for childhood metabolic syndrome^34^, we defined elevated BP as BP ≥75th percentile, which afforded us adequate statistical power. We also conducted sensitivity analyses using a cutoff of SBP ≥90th percentile or ≥120 mmHg, or a cutoff of SBP/DBP ≥90th percentile or ≥120/80 mmHg^11^, and the associations were similar. (See Supplemental Tables).

### Major Covariates

We ascertained maternal pre-pregnancy height and weight by from a maternal questionnaire collected at an interview within 2 to 3 days of delivery, used this to calculate maternal pre-pregnancy BMI. In a subset of our study population (N = 672) we found that BMI determined from self-reported height and weight showed a high level of agreement with BMI taken from medical records (r = 0.89, P < 0.001)^36^. We classified maternal race and ethnicity as Black, Hispanic, Haitian, or Other (White, Asian, Pacific Islander, and mothers who reported more than one race)^37^.

We defined the following maternal and pregnancy-related outcomes using medical records. We classified maternal diabetes status as nondiabetic or diabetic (either pre-existing or gestational diabetes)^30^. We defined maternal hypertensive disorders in pregnancy^38^ as pregnancy-induced hypertension (i.e., gestational hypertension, preeclampsia, eclampsia and Hemolysis, Elevated Liver enzymes, Low Platelet count (HELLP) syndrome) or hypertension that existed prior to pregnancy (referred to here as existing hypertension). We determined gestational age by the first day of the last menstrual period and early prenatal ultrasonographic results^31^. We further categorized births into term (≥37 wks) and preterm (<37 wks).

We categorized birthweight for gestational age (BW-GA) into three groups: small for gestational age (SGA; <10th percentile), appropriate for gestational age (AGA; 10th to 90th percentile), and large for gestational age (LGA; >90th percentile) according to an established local sex-and-race-specific reference population^39^. We prefer the local standard because our population is enriched by African American (41.4%) and thus differs from from the National reference cohort^40^. Nevertheless, our results were similar when we used alternative national BW-GA standards^40^. Birthweight was also categorized into low birthweight (<2500 g) and normal birthweight (≥2500 g).

### Statistical Analysis

Our primary outcome variable was SBP measured at the last well-child visit, which we modeled as SBP percentile (continuous variable) and elevated SBP (SBP ≥75th percentile vs. SBP <75^th^ percentile). Our primary predictor variables were BW-GA categories and nighttime sleep duration.

We used non-parametric regression smoothing plots to explore the tracking of blood pressure in early childhood^41,42^. This tracking analysis was limited to 1026 children who had at least 2 pediatric well child visits with BP measurements with the earliest visit at 3 years of age. Associations were similar in males and females (Supplementary Figure S2), and thus we pooled both sexes for all analyses. We further examined tracking of BP according to BW-GA categories (SGA, AGA, and LGA). Nighttime sleep duration tertiles were specific for age (based on sleep questionnaire visit age) and sex because of age and sex differences in sleep duration in childhood^43^ (Supplementary Table S10). For ease of interpretation, tertile 1 (T1) represents “short” sleep duration; tertile 2 (T2) represents “medium” sleep duration; and tertile 3 (T3) represents “long” sleep duration (Supplementary Table S10). Trends were similar when we used alternative national sleep duration categories^44^.

We next estimated the individual and joint association of BW-GA categories and sleep duration with child BP percentiles (using linear regression models) and childhood elevated BP (using log-Poisson models). Poisson regression models with robust variance (an approximation to log binomial regression models) were used to estimate adjusted prevalence ratios and 95% confidence intervals. We evaluated whether child sleep duration modified the association of BW-GA with childhood SBP by including a cross-product term for sleep duration status with BW-GA category in our multivariable models for SBP.

To address threats to validity from confounding, we adjusted Model 1 for sex, age, maternal race and ethnicity, pre-pregnancy BMI, hypertensive disorders in pregnancy, and diabetes in pregnancy. Covariates included in Model 1 were selected based on previous literature documenting their association with BW-GA, childhood sleep duration and childhood blood pressure. In the final model (Model 2), we additionally adjusted for child current BMI z-score (based on CDC growth charts^31^), which may be on the causal pathway and thus constitute a potential mediator. To explore the influence of socioeconomic indicators, we have performed additional analyses with models that further included maternal education and the total year household income. We have presented the results from these models in Supplemental Tables.

To further assess the robustness of the findings, we conducted sensitivity analyses restricted to participants who have 2 or more BP measurements during childhood using the generalized estimating equation (GEE). We also performed sensitivity analyses of children (n = 441) who had their sleep ascertained between the ages of 4 and 9 years. Statistical analyses were performed using SAS (SAS Institute), version 9.4, and statistical tests were two-sided with significance defined at P < 0.05.

### Data availability

The datasets generated during and/or analyzed during the current study are available from the corresponding author on reasonable request.

## Electronic supplementary material

Supplemental tables and figures
