# Supplementary material for: The Joint Association of Small for Gestational Age and Nighttime Sleep with Blood Pressure in Childhood
Source: Sci Rep. 2018 Jun 25;8:9632. doi: 10.1038/s41598-018-27815-1 (PMC6018546; doi:10.1038/s41598-018-27815-1)
Supplement: Supplementary file 1 — Supplemental tables and figures [file 41598_2018_27815_MOESM1_ESM.docx]

The Joint Association of Small for Gestational Age and Nighttime Sleep with Blood Pressure in Childhood

Hongjian Wang MD. PhD^1,2^, Noel Mueller PhD, MPH^3,4^, Guoying Wang MD, PhD^2^, Xiumei Hong, MD, PhD^2^, Ting Chen MS^2^, Yuelong Ji MS^2^, Colleen Pearson BA^5^, Lawrence J. Appel, MD^3,4^, Xiaobin Wang MD, MPH, ScD^2,6.^

**Online- only supplemental material**

Figure S1. Flow diagram of Boston Birth Cohort participants included in final analytic data set.

Figure S2 Tracking of Blood pressure in early childhood. (N=1026)

Table S1 The individual associations of birth weight for gestational age and child sleep duration with child SBP percentile and elevated SBP (SBP ≥75%) in children from the Boston Birth Cohort.

Table S2 The combined association of birth weight for gestational age and child sleep duration on child SBP percentile and elevated SBP (SBP ≥75%) in children from the Boston Birth Cohort.

Table S3 The individual associations of birth weight for gestational age and child sleep duration with child DBP percentile and elevated DBP (DBP ≥75%) in children from the Boston Birth Cohort.

Table S4 The combined association of birth weight for gestational age and child sleep duration on child DBP percentile and elevated DBP (DBP ≥75%) in children from the Boston Birth Cohort

Table S5. The individual associations of birth weight for gestational age and child sleep duration with child SBP percentile and elevated SBP (SBP ≥75%) in children from the Boston Birth Cohort (age range: 4-9 years; sample size:441)

Table S6 The combined association of birth weight for gestational age and child sleep duration on child SBP percentile and elevated SBP (SBP ≥75%) in children from the Boston Birth Cohort (age range: 4-9 years ; sample size:441)

Table S7 The individual associations of birth weight for gestational age and child sleep duration with child SBP percentile and elevated BP (a cutoff of SBP ≥90th percentile or ≥120mmHg, or a cutoff of SBP/DBP≥90th percentile or ≥120/80mmHg) in children from the Boston Birth Cohort

Table S8 The combined association of birth weight for gestational age and child sleep duration on child SBP percentile and elevated BP (a cutoff of SBP ≥90th percentile or ≥120mmHg, or a cutoff of SBP/DBP≥90th percentile or ≥120/80mmHg) in children from the Boston Birth Cohort

Table S9 Comparison of Prenatal and Early Childhood Characteristics between Included and Excluded Study Participants

Table S10 Sleep duration tertiles (T) on each age group by sex.

**Figure S1. Flow diagram of Boston Birth Cohort participants included in final analytic data set**

3,098 mother-infant pairs under follow-up at Boston Medical Center (BMC) between 2003-2014

1,212 were excluded:

- 366 didn’t have their first BP measured (born after January 2011)

- 25 had first BP measured after 10 years

-814 did not have blood pressure measured between 3-9 years

-4 did not have electronic medical data

- 3 had missing data on age

1886 children were eligible for postnatal follow-up at BMC and had blood pressure measured at 3-9 years of age

708 had missing values on key covariates:

-606 did not have or only have sleep data ascertained after their blood pressure measurements were taken

-102 children’s mothers did not have prepregnancy BMI

1178 mothers and children were in our final analytic sample

**Figure S2 Tracking of Blood pressure in early childhood. (N=1026)**


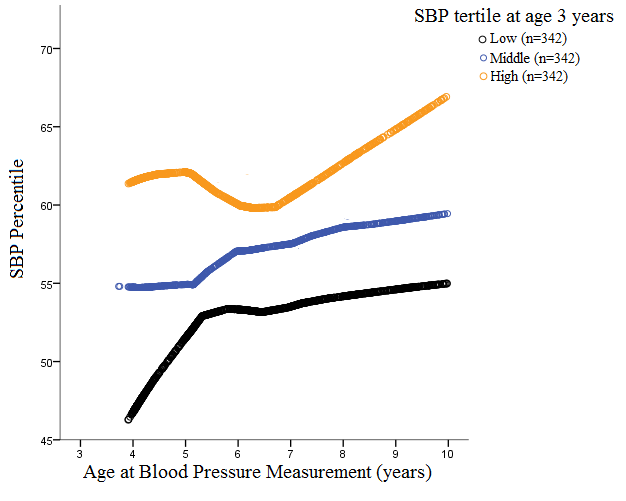


|  |
| --- |
|  |

| Boys | Girls |
| --- | --- |
| 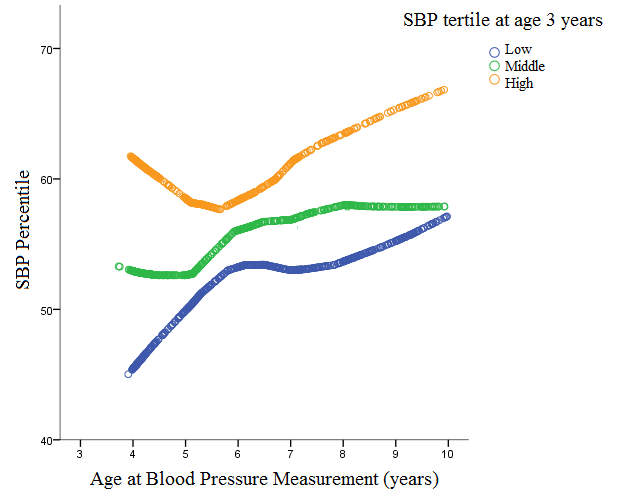 | 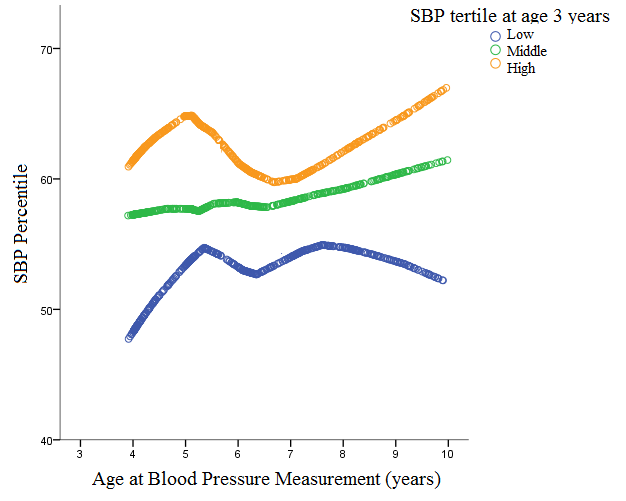 |
|  |  |
|  |  |

Y-axis is the mean of SBP percentiles. X-axis is the age at blood pressure measurement (the age when blood pressure was measured in well child visit). The participants were grouped as low, middle and high tertile based on SBP percentile measured at age 3. The median (interquartile) SBP percentile at age 3 was SBP: 23.7 (13.3–32.9) for lowest tertile; SBP: 56.3(47.7-64.2) for middle tertile; and SBP: 84.5(76.0-92.1) for highest tertile.

**Table S1. The individual associations of birth weight for gestational age and child sleep duration with child SBP percentile and elevated SBP (SBP ≥75%) in children from the Boston Birth Cohort (BBC).**

|  |  | **Child SBP percentile** | | | **Child elevated SBP** | | |
| --- | --- | --- | --- | --- | --- | --- | --- |
|  |  |  | **Model 1** | **Model 2** |  | **Model 1** | **Model 2** |
|  | **N** | **Mean (SD)** | **ß (95%CI)** | **ß (95%CI)** | **Case, No (%)** | **RR (95%CI)** | **RR (95%CI)** |
| ***Birthweight for gestational age^a^*** | | | | |  |  |  |
| LGA | 121 | 54.0 (25.3) | 0 |  | 27 (22.3) | 1 |  |
| AGA | 903 | 56.8 (25.0) | 4.70 (-0.09,9.50) | 4.79(-0.01,9.59) | 257 (28.5) | 1.41 (1.00,2.00) | 1.43(1.01,2.02)* |
| SGA | 154 | 59.5 (24.6) | 7.78 (1.74,13.82)* | 7.69(1.65,13.73)* | 51 (33.1) | 1.65 (1.10,2.47)* | 1.64 (1.09,2.45)* |
| BW-GA | |  | -1.44(-2.82,-0.05)* | -1.40(-2.79,-0.01)* |  | 0.92(0.84,1.00) | 0.92(0.85,1.01) |
| ***Sleep duration tertiles ^b^*** | | | |  |  |  |  |
| Short (T1) | 386 | 56.9 (25.8) | 0 |  | 114 (29.5) | 1 |  |
| Medium (T2) | 402 | 58.4 (24.4) | 1.40 (-2.06,4.86) | 1.36(-2.10,4.82) | 119 (29.6) | 1.01 (0.82,1.26) | 1.01(0.81.1.25) |
| Long (T3) | 390 | 55.3 (24.7) | -1.33 (- 4.82,2.16) | -1.42(-4.91,2.07) | 102 (26.2) | 0.90 (0.72,1.13) | 0.89(0.71,1.12) |
| 1-hour increment in sleep duration (h) | | | -0.78(-1.87,0.31) | -0.82(-1.91,0.27) |  | 0.95(0.88,1.02) | 0.95(0.88,1.02) |

Model 1: Adjusted by sex, age, race/ethnicity (Black, Hispanic, Haitian, others), maternal pre-pregnancy BMI (continuous), maternal hypertensive disorder (yes vs. no) and maternal diabetes (yes vs. no); Model 2: Model 1 with maternal education (no school or elementary school; some secondary school (9th grade and above); completed high school or General Educational Development (GED); some college; college degree and above; unknown) and the total year household income (<$5,000/$5000-9,999; $10,000-14,999; $15,000-19,999; $20,000-24,999; $25,000-29,999; $30,000-34,999; $35,000-39,999; $40,000-49,999; $50,000-59,999; $60,000 and over; don't know); *P<0.05; †P<0.01; a: additional adjustment for sleep duration; b: additional adjustment for birth weight for gestational age; BW-GA: Birthweight for gestational age;

**Table S2 The combined association of birth weight for gestational age and child sleep duration on child SBP percentile and elevated SBP (SBP ≥75%) in children from the Boston Birth Cohort (BBC)**

|  |  | **Child SBP percentile** | |  | **Child elevated SBP** | |  |
| --- | --- | --- | --- | --- | --- | --- | --- |
|  |  |  | **Model 1** | **Model 2** |  | **Model 1** | **Model 2** |
|  | **N** | **Mean (SD)** | **ß (95%CI)** | **ß (95%CI)** | **Case, No (%)** | **RR (95%CI)** | **RR (95%CI)** |
| ***Combined effect of birth weight for gestational age and sleep duration on blood pressure*** | | | | | | |  |
| ***SGA*** | | |  |  |  |  |  |
| Short (T1) | 39 | 67.8 (25.5) | 0 |  | 20 (51.3) | 1 |  |
| Medium (T2) | 60 | 57.3 (25.3) | -10.07 (-20.00,-0.15)* | -10.09(-20.00,-0.19)* | 19 (31.7) | 0.63 (0.40, 1.02) | 0.64(0.40,1.03) |
| Long (T3) | 55 | 56.1 (22.0) | -11.34 (-21.44, -1.24)* | -11.35(-21.44,-1.26)* | 12 (21.8) | 0.43 (0.24,0.76)^†^ | 0.43(0.24,0.76)^†^ |
| ***AGA*** |  |  |  |  |  |  |  |
| Short (T1) | 305 | 55.7 (25.4) | -12.29 (-20.51,-4.08) ^†^ | -12.00(-20.22,-3.78)^†^ | 82 (26.9) | 0.53 (0.37,0.75)^†^ | 0.54(0.38,0.76)^†^ |
| Medium (T2) | 294 | 59.3 (24.2) | -8.79 (-17.03,-0.55)* | -8.56(-16.81,-0.31)* | 91 (31.0) | 0.61 (0.43,0.86)^†^ | 0.62(0.44,0.88)^†^ |
| Long (T3) | 304 | 55.5 (25.1) | -12.16 (-20.37, -3.94) ^†^ | -12.00(-20.22,-3.79)^†^ | 84 (27.6) | 0.56 (0.39,0.79)^†^ | 0.56(0.39,0.80)^†^ |
| ***LGA*** |  |  |  |  |  |  |  |
| Short (T1) | 42 | 55.4 (27.1) | -14.69(-25.52,-3.86) ^†^ | -14.53(-25.36,-3.70)^†^ | 12 (28.6) | 0.49 (0.27,0.87)* | 0.49(0.28,0.88)* |
| Medium (T2) | 48 | 53.7 (23.9) | -15.75(-26.23,-5.28) ^†^ | -15.79(-26.27,-5.31)^†^ | 9 (18.8) | 0.35 (0.18,0.67)^†^ | 0.34(0.18,0.66)^†^ |
| Long (T3) | 31 | 52.6 (25.6) | -16.53(-28.20,-4.86)^†^ | -16.28(-27.95,-4.61)^†^ | 6 (19.4) | 0.38 (0.17, 0.81) * | 0.39(0.18,0.83)* |
| ***Stratified by birth weight for gestational age*** | | | |  |  |  |  |
| ***SGA*** |  |  |  |  |  |  |  |
| Short (T1) | 39 | 67.8 (25.5) | 0 |  | 20 (51.3) | 1 |  |
| Medium (T2) | 60 | 57.3 (25.3) | -10.43 (-19.91,-0.95)* | -10.58(-20.07,-1.10)* | 19 (31.7) | 0.62 (0.37,1.03) | 0.61(0.37,1.03) |
| Long (T3) | 55 | 56.1 (22.0) | -12.28 (-22.00,-2.57)* | -12.21(-21.94,-2.47)* | 12 (21.8) | 0.44 (0.25,0.79)^†^ | 0.45(0.25,0.79)^†^ |
| 1-hour increment in sleep duration (h) | | | -3.74(-7.12,-0.36)* | -3.78(-7.17,-0.38)* |  | 0.76(0.63,0.91)^†^ | 0.75(0.62,0.90)^†^ |
| ***AGA*** |  |  |  |  |  |  |  |
| Short (T1) | 305 | 55.7 (25.4) | 0 |  | 82 (26.9) | 1 |  |
| Medium (T2) | 294 | 59.3 (24.2) | 3.49 (-0.48,7.45) | 3.47(-0.49,7.43) | 91 (31.0) | 1.15 (0.90,1.48) | 1.15(0.90,1.48) |
| Long (T3) | 304 | 55.5 (25.1) | 0.17 (-3.77, 4.12) | 0.07(-3.88,4.02) | 84 (27.6) | 1.06 (0.82,1.37) | 1.05(0.81,1.36) |
| 1-hour increment in sleep duration (h) | | | -0.48 (-1.68,0.72) | -0.52(-1.72,0.68) |  | 0.99(0.91,1.07) | 0.99(0.91,1.07) |
| ***LGA*** |  |  |  |  |  |  |  |
| Short (T1) | 42 | 55.4 (27.1) | 0 |  | 12 (28.6) | 1 |  |
| Medium (T2) | 48 | 53.7 (23.9) | -2.39 (-12.21, 7.43) | -1.72(-11.59,8.14) | 9 (18.8) | 0.64 (0.30,1.38) | 0.65(0.30,1.38) |
| Long (T3) | 31 | 52.6 (25.6) | -0.25 (-11.20, 10.70) | -0.33(-11.25,10.60) | 6 (19.4) | 0.86 (0.38,1.94) | 0.85(0.37,1.97) |
| 1-hour increment in sleep duration (h) | | | 0.18 (-3.82, 4.17) | 0.28(-3.71,4.28) |  | 0.89(0.66,1.20) | 0.89(0.65,1.21) |

Model 1: adjusted for sex, age, race/ethnicity (Black, Hispanic, Haitian, others), maternal pre-pregnancy BMI (continuous), maternal hypertensive disorder (yes vs. no) and maternal diabetes (yes vs. no); Model 2: Model 1 with maternal education (no school or elementary school; some secondary school (9th grade and above); completed high school or General Educational Development (GED); some college; college degree and above; unknown) and the total year household income (<$5,000/$5000-9,999; $10,000-14,999; $15,000-19,999; $20,000-24,999; $25,000-29,999; $30,000-34,999; $35,000-39,999; $40,000-49,999; $50,000-59,999; $60,000 and over; don't know); *P<0.05; †P<0.01; P for interaction between birthweight for gestational age and sleep duration on elevated SBP: 0.0056. P for interaction between birthweight for gestational age and sleep duration on SBP percentiles: 0.087;

**Table S3 The individual associations of birth weight for gestational age and child sleep duration with child DBP percentile and elevated DBP (DBP ≥75%) in children from the Boston Birth Cohort.**

|  |  | **Child DBP percentile** | | | **Child elevated DBP** | | |
| --- | --- | --- | --- | --- | --- | --- | --- |
|  |  |  | **Model 1** | **Model 2** |  | **Model 1** | **Model 2** |
|  | **N** | **Mean (SD)** | **ß (95%CI)** | **ß (95%CI)** | **Case, No (%)** | **RR (95%CI)** | **RR (95%CI)** |
| ***Birthweight for gestational age^a^*** | | | | |  |  |  |
| LGA | 121 | 66.6(20.1) | 0 | 0 | 48(39.7) | 1 |  |
| AGA | 903 | 66.9(20.6) | 1.49(-2.33,5.30) | 1.75(-2.07,5.58) | 368(40.8) | 1.02(0.95,1.09) | 1.02(0.95,1.09) |
| SGA | 154 | 68.1(18.7) | 3.79(-1.01,8.59) | 4.39 (-0.47,9.26) | 65(42.2) | 1.04(0.96,1.13) | 1.04(0.95,1.13) |
| BW-GA |  |  | -1.15(-2.25,-0.05)* | -1.37(-2.49,-0.25)* |  | 0.99(0.97,1.00) | 0.98(0.97,1.00) |
| ***Sleep duration tertiles ^b^*** | | | |  |  |  |  |
| Short (T1) | 386 | 66.3(21.3) | 0 |  | 158(40.9) | 1 |  |
| Medium (T2) | 402 | 67.3(20.0) | 0.63(-2.12,3.38) | 0.97(-1.79,3.72) | 163(40.6) | 1.00(0.95, 1.04) | 1.00(0.95, 1.05) |
| Long (T3) | 390 | 67.4(19.5) | 1.25(-1.53,4.02) | 1.62(-1.16,4.40) | 160(41.0) | 1.00(0.96, 1.05) | 1.01(0.96, 1.06) |
| 1-hour increment in sleep duration (h) | | | -0.02(-0.89,0.85) | 0.09 (-0.78,0.96) |  | 0.99(0.97, 1.00) | 0.99(0.97,1.00) |

Model 1: Adjusted by sex, age, race/ethnicity (Black, Hispanic, Haitian, others), maternal pre-pregnancy BMI (continuous), maternal hypertensive disorder (yes vs. no) and maternal diabetes (yes vs. no); Model 2: Model 1 with children BMI z score; *P<0.05; †P<0.01; a: additional adjustment for sleep duration; b: additional adjustment for birth weight for gestational age; BW-GA: Birthweight for gestational age;

**Table S4 The combined association of birth weight for gestational age and child sleep duration on child DBP percentile and elevated DBP (DBP ≥75%) in children from the Boston Birth Cohort**

|  |  | **Child DBP percentile** | |  | **Child elevated DBP** | |  |
| --- | --- | --- | --- | --- | --- | --- | --- |
|  |  |  | **Model 1** | **Model 2** |  | **Model 1** | **Model 2** |
|  | **N** | **Mean (SD)** | **ß (95%CI)** | **ß (95%CI)** | **Case, No (%)** | **RR (95%CI)** | **RR (95%CI)** |
| ***Combined effect of birth weight for gestational age and sleep duration on blood pressure*** | | | | | | |  |
| *SGA* |  |  |  |  |  |  |  |
| Short (T1) | 39 | 70.3(18.4) | 0 |  | 18(46.2) | 1 |  |
| Medium (T2) | 60 | 66.6(19.7) | -2.69 (-10.59,5.21) | -2.33(-10.27,5.61) | 23(38.3) | 0.96(0.83,1.10) | 0.97(0.84,1.11) |
| Long (T3) | 55 | 68.1(17.9) | -1.36 (-9.41,6.68) | -0.74(-8.84,7.35) | 24(43.6) | 0.99(0.86,1.14) | 1.00(0.87,1.15) |
| *AGA* |  |  |  |  |  |  |  |
| Short (T1) | 305 | 66.1(21.7) | -4.77(-11.31,1.78) | -5.02(-11.65,1.60) | 126(41.3) | 0.96(0.86,1.08) | 0.96(0.86, 1.09) |
| Medium (T2) | 294 | 67.6(20.2) | -3.37(-9.94, 3.19) | -3.27(-9.91,3.37) | 121(41.2) | 0.96(0.85,1.08) | 0.97(0.86, 1.09) |
| Long (T3) | 304 | 67.0(19.8) | -3.55(-10.10,3.00) | -3.47(-10.09,3.16) | 121(39.8) | 0.96(0.85,1.07) | 0.96(0.86, 1.09) |
| *LGA* |  |  |  |  |  |  |  |
| Short (T1) | 42 | 64.8(21.0) | -6.38(-15.01,2.25) | -6.83(-15.55,1.88) | 14(33.3) | 0.91(0.78,1.06) | 0.91(0.78, 1.07) |
| Medium (T2) | 48 | 65.7(20.0) | -7.23(-15.57,1.12) | -7.50(-15.92,0.92) | 19(39.6) | 0.93(0.80,1.07) | 0.94(0.81, 1.09) |
| Long (T3) | 31 | 70.3(19.1) | -1.45(-10.74,7.85) | -1.60(-10.94,7.75) | 15(48.4) | 1.00(0.85,1.18) | 1.01(0.86, 1.19) |
| ***Stratified by birth weight for gestational age*** | | | | |  |  |  |
| *SGA* |  |  |  |  |  |  |  |
| Short (T1) | 39 | 70.3(18.4) | 0 |  | 18(46.2) | 1 |  |
| Medium (T2) | 60 | 66.6(19.7) | -3.83(-11.10,3.45) | -3.36(-10.55,3.83) | 23(38.3) | 0.94(0.82,1.08) | 0.95(0.83,1.09) |
| Long (T3) | 55 | 68.1(17.9) | -3.02(-10.47,4.44) | -3.14(-10.52,4.24) | 24(43.6) | 0.97(0.84,1.11) | 0.97(0.84,1.11) |
| 1-hour increment in sleep duration (h) | | | -0.77(-3.35,1.82) | -0.91(-3.47,1.64) |  | 1.00(0.95,1.05) | 1.00(0.95,1.05) |
| *AGA* |  |  |  |  |  |  |  |
| Short (T1) | 305 | 66.1(21.7) | 0 |  | 126(41.3) | 1 |  |
| Medium (T2) | 294 | 67.6(20.2) | 1.41(-1.77,4.59) | 1.78(-1.40,4.95) | 121(41.2) | 1.00(0.95,1.05) | 1.01(0.95,1.06) |
| Long (T3) | 304 | 67.0(19.8) | 1.29(-1.88,4.45) | 1.59(-1.56,4.75) | 121(39.8) | 0.99(0.94,1.05) | 1.00(0.95,1.06) |
| 1-hour increment in sleep duration (h) | | | -0.07(-1.03,0.89) | 0.03(-0.92,0.99) |  | 0.98(0.97,1.00) | 0.99(0.97,1.00) |
| *LGA* |  |  |  |  |  |  |  |
| Short (T1) | 42 | 64.8(21.0) | 0 |  | 14(33.3) | 1 |  |
| Medium (T2) | 48 | 65.7(20.0) | -2.29(-10.06,5.47) | -2.68(-10.44,5.08) | 19(39.6) | 1.00(0.87,1.15) | 1.00(0.86,1.14) |
| Long (T3) | 31 | 70.3(19.1) | 5.57(-3.09,14.24) | 5.02(-3.67,13.70) | 15(48.4) | 1.11(0.95,1.30) | 1.10(0.94,1.29) |
| 1-hour increment in sleep duration (h) | | | 1.58(-1.60,4.77) | 1.42(-1.76,4.61) |  | 1.01(0.95,1.07) | 1.01(0.95,1.07) |

Model 1: adjusted for sex, age, race/ethnicity (Black, Hispanic, Haitian, others), maternal pre-pregnancy BMI (continuous), maternal hypertensive disorder (yes vs. no) and maternal diabetes (yes vs. no); Model 2: Model 1 with children BMI z score; *P<0.05; †P<0.01; P for interaction between birthweight for gestational age and sleep duration on elevated DBP: 0.553. P for interaction between birthweight for gestational age and sleep duration on DBP percentiles: 0.662;

**Table S5. The individual associations of birth weight for gestational age and child sleep duration with child SBP percentile and elevated SBP (SBP ≥75%) in children from the Boston Birth Cohort (age range: 4-9 years; sample size:441)**

|  |  | **Child SBP percentile** | | | **Child elevated SBP** | | |
| --- | --- | --- | --- | --- | --- | --- | --- |
|  |  |  | **Model 1** | **Model 2** |  | **Model 1** | **Model 2** |
|  | **N** | **Mean (SD)** | **ß (95%CI)** | **ß (95%CI)** | **Case, No (%)** | **RR (95%CI)** | **RR (95%CI)** |
| ***Birthweight for gestational age^a^*** | | | | |  |  |  |
| LGA | 48 | 55.6 (26.4) | 0 |  | 11 (22.9) | 1 |  |
| AGA | 333 | 57.6 (25.3) | 4.07(-3.84,11.98) | 5.66 (-2.13,13.35) | 103 (30.9) | 1.63 (0.93,2.84) | 1.78(1.02,3.12)* |
| SGA | 60 | 64.2 (24.4) | 10.47(0.54,20.39)* | 13.79 (3.95,23.62) † | 27 (45.0) | 2.28 (1.24,4.21)* | 2.60 (1.41,4.78)† |
| BW-GA |  |  | -1.80 (-4.10, 0.50) | -2.80 (-5.10, -0.50)* |  | 0.86 (0.76, 0.98)* | 0.84(0.74, 0.95) † |
| ***Sleep duration tertiles^b^*** | | | |  |  |  |  |
| Short (T1) | 138 | 58.0 (25.4) | 0 |  | 41(29.7) | 1 |  |
| Medium (T2) | 155 | 60.5 (24.9) | 1.64 (-4.16,7.45) | 2.33(-3.40,8.06) | 55(35.5) | 1.13(0.81,1.58) | 1.14(0.82,1.58) |
| Long (T3) | 148 | 56.3 (25.8) | -2.14 (- 8.00,3.72) | -1.12(-6.91,4.67) | 45(30.4) | 1.01(0.71,1.44) | 1.03(0.73,1.46) |
| 1-hour increment in sleep duration (h) | | | -1.27(-3.35,0.82) | -0.82(-2.89,1.24) |  | 0.94(0.84,1.05) | 0.95(0.85,1.06) |

Model 1: Adjusted by sex, age, race/ethnicity (Black, Hispanic, Haitian, others), maternal pre-pregnancy BMI (continuous), maternal hypertensive disorder (yes vs. no) and maternal diabetes (yes vs. no); Model 2: Model 1 with children BMI z score; *P<0.05; †P<0.01; a: additional adjustment for sleep duration; b: additional adjustment for birth weight for gestational age; a BW-GA: Birthweight for gestational age;

**Table S6 The combined association of birth weight for gestational age and child sleep duration on child SBP percentile and elevated SBP (SBP ≥75%) in children from the Boston Birth Cohort (age range: 4-9 years; sample size:441)**

|  |  | **Child SBP percentile** | |  | **Child elevated SBP** | |  |
| --- | --- | --- | --- | --- | --- | --- | --- |
|  |  |  | **Model 1** | **Model 2** |  | **Model 1** | **Model 2** |
|  | **N** | **Mean (SD)** | **ß (95%CI)** | **ß (95%CI)** | **Case, No (%)** | **RR (95%CI)** | **RR (95%CI)** |
| ***Combined effect of birth weight for gestational age and sleep duration on blood pressure*** | | | | | |  |  |
| *SGA* |  |  |  |  |  |  |  |
| Short (T1) | 11 | 76.0(24.6) | 0 |  | 8(72.7) | 1 |  |
| Medium (T2) | 26 | 61.9(24.5) | -15.79 (-33.33,1.75) | -15.63(-32.81,1.55) | 11(42.3) | 0.54 (0.30, 0.97)* | 0.55(0.31,0.97)* |
| Long (T3) | 23 | 61.1(23.4) | -15.36 (-33.21,2.50) | -14.20(-31.69,3.30) | 8(34.8) | 0.47 (0.25,0.88)* | 0.47(0.26,0.88)* |
| *AGA* |  |  |  |  |  |  |  |
| Short (T1) | 109 | 57.3(24.2) | -19.38(-34.80,-3.96)* | -21.35(-36.49,-6.20)^†^ | 28(25.7) | 0.35 (0.22,0.56)^†^ | 0.33(0.21,0.53) ^†^ |
| Medium (T2) | 114 | 60.7(25.2) | -16.29(-31.72,-0.86)* | -17.40(-32.52,-2.27)* | 41(36.0) | 0.48 (0.31,0.74) ^†^ | 0.47(0.30,0.72) ^†^ |
| Long (T3) | 110 | 54.8(26.4) | -21.96(-37.45,-6.47) ^†^ | -23.00 (-38.19,-7.82)^†^ | 34(30.9) | 0.42 (0.27,0.67) ^†^ | 0.41(0.26,0.65) ^†^ |
| *LGA* |  |  |  |  |  |  |  |
| Short (T1) | 18 | 51.2(29.4) | -29.00 (-48.03, -9.97)^†^ | -32.59(-51.29,-13.88)^†^ | 5(27.8) | 0.29 (0.12,0.70) ^†^ | 0.25(0.10,0.62) ^†^ |
| Medium (T2) | 15 | 56.7(24.9) | -21.09(-40.72,-1.46)* | -24.37(-43.65,-5.09)* | 3(20.0) | 0.23 (0.08,0.67) ^†^ | 0.20(0.07,0.57) ^†^ |
| Long (T3) | 15 | 59.8(25.2) | -18.84(-38.35, 0.66) | -20.91(-40.04,-1.78)* | 3(20.0) | 0.25 (0.08, 0.74) * | 0.23(0.08,0.67) ^†^ |
| ***Stratified by birth weight for gestational age*** | | | |  |  |  |  |
| *SGA* |  |  |  |  |  |  |  |
| Short (T1) | 11 | 76.0(24.6) | 0 |  | 8(72.7) | 1 |  |
| Medium (T2) | 26 | 61.9(24.5) | -16.42(-31.90,-0.94)* | -16.36(-31.62,-1.10)* | 11(42.3) | 0.51 (0.26,1.01) | 0.54(0.27,1.06) |
| Long (T3) | 23 | 61.1(23.4) | -16.54(-32.16,-0.93)* | -16.13(-31.54,-0.72)* | 8(34.8) | 0.42 (0.20,0.89)* | 0.44(0.20,0.94)* |
| 1-hour increment in sleep duration (h) | | | -5.31(-10.46,-0.16)* | -5.07(-10.18,0.03) |  | 0.74(0.57,0.97)* | 0.76(0.57,1.00) * |
| *AGA* |  |  |  |  |  |  |  |
| Short (T1) | 109 | 57.3(24.2) | 0 |  | 28(25.7) | 1 |  |
| Medium (T2) | 114 | 60.7(25.2) | 2.96 (-3.64,9.56) | 4.00(-2.47,10.46) | 41(36.0) | 1.36(0.91,2.02) | 1.37(0.92,2.03) |
| Long (T3) | 110 | 54.8(26.4) | -2.67 (-9.36, 4.02) | -1.42(-7.96,5.13) | 34(30.9) | 1.21(0.79,1.85) | 1.24(0.81,1.87) |
| 1-hour increment in sleep duration (h) | | | -1.44(-3.82,0.95) | -1.04(-3.38,1.30) |  | 1.00(0.88,1.13) | 1.00(0.88,1.14) |
| *LGA* |  |  |  |  |  |  |  |
| Short (T1) | 18 | 51.2(29.4) | 0 | 0 | 5(27.8) | 1 |  |
| Medium (T2) | 15 | 56.7(24.9) | 6.02 (-11.96,24.00) | 6.18(-12.14,24.50) | 3(20.0) | 0.54 (0.11,2.57) | 0.54(0.11,2.71) |
| Long (T3) | 15 | 59.8(25.2) | 12.09(-4.96,29.14) | 12.18(-4.99,29.35) | 3(20.0) | 0.98 (0.27,3.53) | 0.93(0.23,3.81) |
| 1-hour increment in sleep duration (h) | | | 5.09(-1.79,11.97) | 5.29(-1.77,12.34) |  | 0.73(0.42,1.26) | 0.73(0.43,1.25) |

Model 1: adjusted for sex, age, race/ethnicity (Black, Hispanic, Haitian, others), maternal pre-pregnancy BMI (continuous), maternal hypertensive disorder (yes vs. no) and maternal diabetes (yes vs. no); Model 2: Model 1 with children BMI z score; *P<0.05; †P<0.01; P for interaction between birthweight for gestational age and sleep duration on elevated SBP: 0.120. P for interaction between birthweight for gestational age and sleep duration on SBP percentiles: 0.410;

**Table S7 The individual associations of birth weight for gestational age and child sleep duration with child SBP percentile and elevated BP (a cutoff of SBP ≥90th percentile or ≥120mmHg, or a cutoff of SBP/DBP≥90th percentile or ≥120/80mmHg) in children from the Boston Birth Cohort**

|  |  | **≥90^th^ SBP or SBP≥120** | | **SBP or DBP ≥90th percentile or**  **SBP/DBP ≥120/80 mm Hg** | |
| --- | --- | --- | --- | --- | --- |
|  | **N** | **Case, No (%)** | **RR (95%CI)** | **Case, No (%)** | **RR (95%CI)** |
| ***Birthweight for gestational age^a^*** |  |  |  |  |  |
| LGA | 121 | 8(6.6) | 1 | 15(12.4) | 1 |
| AGA | 903 | 96(10.6) | 1.04(1.00,1.09) | 163(18.1) | 1.06(1.00, 1.12) |
| SGA | 154 | 23(14.9) | 1.09(1.02,1.16)* | 31(20.1) | 1.09(1.01,1.17)* |
| BW-GA |  |  | 0.99(0.97,1.00) |  | 0.99 (0.97, 1.00) |
| ***Sleep duration tertiles ^b^*** |  |  |  |  |  |
| Short (T1) | 386 | 45(11.7) | 1 | 72(18.7) | 1 |
| Medium (T2) | 402 | 45(11.2) | 1.00(0.96, 1.04) | 73(18.2) | 1.00(0.95,1.05) |
| Long (T3) | 390 | 37(9.5) | 0.98(0.94,1.02) | 64(16.4) | 0.98(0.94,1.03) |
| 1-hour increment in sleep duration (h) |  |  | 0.99(0.98,1.01) |  | 0.99(0.97,1.00) |

Adjusted by sex, age, race/ethnicity (Black, Hispanic, Haitian, others), maternal pre-pregnancy BMI (continuous), maternal hypertensive disorder (yes vs. no), maternal diabetes (yes vs. no). *P<0.05; †P<0.01; a: additional adjustment for sleep duration; b: additional adjustment for birth weight for gestational age; BW-GA: Birthweight for gestational age;

**Table S8 The combined association of birth weight for gestational age and child sleep duration on child SBP percentile and elevated BP (a cutoff of SBP ≥90th percentile or ≥120mmHg, or a cutoff of SBP/DBP≥90th percentile or ≥120/80mmHg) in children from the Boston Birth Cohort**

|  |  | **≥90^th^ SBP or SBP≥120mmHg** | | **SBP or DBP ≥90th percentile or**  **SBP/DBP ≥120/80 mm Hg** | |
| --- | --- | --- | --- | --- | --- |
|  | **N** | **Case, No (%)** | **RR (95%CI)** | **Case, No (%)** | **RR (95%CI)** |
| ***Combined effect of birth weight for gestational age and sleep duration on blood pressure*** | | | | |  |
| *SGA* |  |  |  |  |  |
| Short (T1) | 39 | 10(25.6) | 1 | 12(30.8) | 1 |
| Medium (T2) | 60 | 7(11.7) | 0.89(0.78,1.02) | 11(18.3) | 0.92(0.80,1.05) |
| Long (T3) | 55 | 6(10.9) | 0.88(0.77,1.00)* | 8(14.6) | 0.88(0.77,1.01) |
| *AGA* |  |  |  |  |  |
| Short (T1) | 305 | 30(9.8) | 0.87(0.77,0.97)* | 54(17.7) | 0.89(0.80,1.00) |
| Medium (T2) | 294 | 35(11.9) | 0.88(0.79,0.99)* | 57(19.4) | 0.91(0.81,1.02) |
| Long (T3) | 304 | 31(10.2) | 0.87(0.78,0.98)* | 52(17.1) | 0.89(0.80,1.00) |
| *LGA* |  |  |  |  |  |
| Short (T1) | 42 | 5(11.9) | 0.88(0.77,1.01) | 6(14.3) | 0.87(0.75,1.01) |
| Medium (T2) | 48 | 3(6.3) | 0.84(0.74,0.96) ^†^ | 5(10.4) | 0.83(0.73,0.95) ^†^ |
| Long (T3) | 31 | 0(0) | 0 | 4(12.9) | 0.86(0.74,1.00) |
| ***Stratified by birth weight for gestational age*** | | |  |  |  |
| *SGA* |  |  |  |  |  |
| Short (T1) | 39 | 10(25.6) | 1 | 12(30.8) | 1 |
| Medium (T2) | 60 | 7(11.7) | 0.88(0.78,1.01) | 11(18.3) | 0.91(0.80, 1.05) |
| Long (T3) | 55 | 6(10.9) | 0.88(0.77,1.00)* | 8(14.6) | 0.88(0.77,1.01) |
| 1-hour increment in sleep duration (h) | |  | 0.96(0.91,1.00) |  | 0.96(0.91,1.00) |
| *AGA* |  |  |  |  |  |
| Short (T1) | 305 | 30(9.8) | 1 | 54(17.7) | 1 |
| Medium (T2) | 294 | 35(11.9) | 1.02(0.98,1.07) | 57(19.4) | 1.02(0.97,1.07) |
| Long (T3) | 304 | 31(10.2) | 1.01(0.97,1.05) | 52(17.1) | 1.00(0.95,1.05) |
| 1-hour increment in sleep duration (h) | |  | 1.00(0.99,1.01) |  | 1.00(0.98,1.01) |
| *LGA* |  |  |  |  |  |
| Short (T1) | 42 | 5(11.9) | 1 | 6(14.3) | 1 |
| Medium (T2) | 48 | 3(6.3) | 0.94(0.86,1.05) | 5(10.4) | 0.94(0.83,1.05) |
| Long (T3) | 31 | 0(0) | 0 | 4(12.9) | 0.99(0.86,1.14) |
| 1-hour increment in sleep duration (h) | |  | 0.95(0.91,0.99) ^†^ |  | 0.96(0.91,1.01) |

Multivariable model adjusted for sex, age, race/ethnicity (Black, Hispanic, Haitian, others), maternal pre-pregnancy BMI (continuous), maternal hypertensive disorder (yes vs. no) , maternal diabetes (yes vs. no); *P<0.05; †P<0.01; P for interaction between birthweight for gestational age and sleep duration on .≥90^th^ SBP or SBP.≥120:0.0385; P for interaction between birthweight for gestational age and sleep duration on SBP or DBP ≥90th percentile or SBP/DBP ≥120/80 mm Hg : 0.088;

**Table S9 Comparison of Prenatal and Early Childhood Characteristics between Included and Excluded Study Participants**

| **Variables** | **Total participants age 3-9 years with blood pressure** | **Participants included in the study** | **Participants excluded from the study** |
| --- | --- | --- | --- |
| No. | 1886 | 1178 | 708 |
| ***Maternal Characteristics*** |  |  |  |
| Race, n. (%) |  |  |  |
| Black | 803 (42.7) | 488 (41.4) | 315 (44.9) |
| Hispanic | 321 (17.1) | 217 (18.4) | 104 (14.8) |
| Haitian | 437(23.2) | 273(23.2) | 164(23.4) |
| Other | 319 (17.0) | 200 (17.0) | 119 (17.0) (Missing=6) |
| Prepregnancy BMI, kg/m2 | 26.8(6.6) | 27.0 (6.7) | 26.5 (6.4) (Missing=102) |
| Maternal Diabetes, n. (%) | 207 (11.0) | 145 (12.3) | 62 (8.9) *(Missing=8) |
| Maternal Hypertensive Disorder, n. (%) | 335 (17.8) | 208 (17.7) | 127 (18.1) (Missing=6) |
| ***Children Characteristics*** |  |  |  |
| Age, years | 6.7 (2.2) | 6.9 (2.1) | 6.3 (2.2) * |
| Boys, n. (%) | 916 (48.7) | 598 (50.8) | 318 (45.3) *(Missing=6) |
| Birthweight (g) | 2936.7 (809.0) | 2938.4 (808.2) | 2933.9 (811.1) (Missing=6) |
| Low birthweight, n. (%) | 473 (25.2) | 290 (24.6) | 183 (26.1) (Missing=6) |
| Gestational age (week) | 37.8 (3.5) | 37.8 (3.4) | 37.8 (3.5) (Missing=7) |
| Preterm birth, n. (%) | 485 (25.8) | 295 (25.0) | 190 (27.1) (Missing=6) |
| BMI z-score | 0.8 (1.3) | 0.8 (1.3) | 0.8 (1.3) (Missing=24) |
| Weight z-score | 0.8 (1.3) | 0.8 (1.3) | 0.8 (1.3) (Missing=2) |

BMI: Body Mass Index; Data are shown as mean (standard deviation, SD) or No. (%). *P<0.05

**Table S10 Sleep duration tertiles (T) on each age group by sex.**

|  | **Sleep duration** | | | | | |
| --- | --- | --- | --- | --- | --- | --- |
|  | **N** | **Short (T1)** | **N** | **Medium (T2)** | **N** | **Long (T3)** |
| Boys |  |  |  |  |  |  |
| 1 years old | 47 | 8.7 (6.0,9.9) | 44 | 10.3 (10.0,10.9) | 51 | 11.9 (11.0,18.0) |
| 2 years old | 45 | 8.9 (7.0,9.7) | 46 | 10.3 (9.8,10.7) | 44 | 11.6 (10.8,13.5) |
| 3 years old | 30 | 9.2 (6.3,9.9) | 31 | 10.2 (10.0,10.6) | 29 | 11.5 (10.6,14.8) |
| 4 years old | 19 | 9.0 (7.7,9.9) | 25 | 10.3 (10.0,10.6) | 22 | 11.3 (10.7,12.3) |
| 5 years old | 14 | 9.1 (8.0,9.8) | 15 | 10.3 (9.9,10.6) | 15 | 11.7 (10.6,13.5) |
| 6 years old | 14 | 9.3 (7.6,9.9) | 15 | 10.2 (9.9,10.6) | 14 | 11.1 (10.7,11.5) |
| 7 years old | 9 | 9.1 (8.7,9.5) | 9 | 10.1 (9.6,10.8) | 10 | 11.7 (11.0,15.4) |
| 8 years old | 11 | 9.0 (8.0,9.6) | 13 | 10.0 (9.6,10.4) | 11 | 11.1 (10.4,12.1) |
| 9 years old | 5 | 8.6 (8.0,9.4) | 6 | 9.7 (9.5,10.0) | 4 | 10.9 (10.1,11.8) |
| Girls |  |  |  |  |  |  |
| 1 years old | 42 | 8.8 (6.3,9.9) | 47 | 10.5 (10.0,11.0) | 42 | 12.2 (11.1,15.4) |
| 2 years old | 54 | 9.2 (4.6,10.0) | 45 | 10.5 (10.1,10.9) | 48 | 11.8 (10.9,14.6) |
| 3 years old | 30 | 9.4 (7.3,10.1) | 34 | 10.8 (10.1,11.0) | 28 | 12.0 (11.1,15.0) |
| 4 years old | 17 | 9.0 (6.2,9.7) | 18 | 10.2 (9.9,10.8) | 18 | 11.6 (10.9,13.6) |
| 5 years old | 15 | 9.0 (7.8,10.0) | 15 | 10.4 (10.0,10.9) | 17 | 11.8 (11.0,14.0) |
| 6 years old | 11 | 9.2 (8.1,9.9) | 13 | 10.3 (10.0,10.7) | 12 | 11.3 (10.9,12.1) |
| 7 years old | 11 | 9.3 (7.9,10.0) | 12 | 10.4 (10.1,10.7) | 12 | 11.5 (10.8,15.0) |
| 8 years old | 5 | 9.0 (8.0,9.5) | 6 | 9.8 (9.5,10.4) | 5 | 11.2 (10.6,12.4) |
| 9 years old | 7 | 8.6 (7.7,9.3) | 8 | 9.8 (9.4,10.2) | 8 | 10.6 (10.3,10.8) |

Sleep duration was shown as mean (min, max)
